# The gut-prostate axis in benign prostatic hyperplasia: systematic review of microbial dysbiosis and pathogenic mechanisms

**DOI:** 10.1186/s12894-025-02003-2

**Published:** 2026-02-02

**Authors:** Yuanzhao Xu, Lingyue An, Jiling Xie, Chenggong Luo, Xiaoxue Huang, Guangheng Luo

**Affiliations:** 1 Graduate School of Medicine, Zunyi Medical University, Zunyi, Guizhou 563006 China; 2 Department of Urology, Guizhou Provincial People’s Hospital, Guiyang, Guizhou 550002 China; 3 The Clinical Medical College, Guizhou Medical University, Guiyang, Guizhou 550004 China; 4 GuiZhou University Medical College, Guiyang, Guizhou 550025 China

**Keywords:** Gut microbiota, Benign prostatic hyperplasia, Systematic review

## Abstract

**Background:**

New evidence shows that gut microbiota dysbiosis may play a crucial role in the development process of benign prostatic hyperplasia (BPH). However, at present, the specific characteristics of the gut microbiota in patients with BPH have not been fully clarified.

**Methods:**

The PubMed, MEDLINE and Web of Science databases were systematically searched to find the clinical and preclinical studies related to the relationship between BPH and gut microbiota from the establishment of the databases to October 7, 2025. And the studies reporting on gut microbiota and BPH were analyzed.

**Results:**

A total of 10 preclinical studies and 6 clinical studies were included. These studies covered 413 patients with BPH, 338 controls, and 5 different types of BPH mouse models in total. Compared with the control group, there were significant differences in β-diversity in the BPH group. A significant increase in the Firmicutes/Bacteroidetes (F/B) ratio was regarded as a marker of the pathological condition. Specifically, changes in the abundances of Prevotella, Ruminococcus, and Lactobacillus may play a key role in the pathogenesis of the occurrence and development of BPH. The imbalance of interleukin-6 (IL-6) and interleukin-18 (IL-18), as well as changes in the levels of intestinal tight junction protein-1 and claudin-1, may also be related to the pathogenesis of BPH.

**Conclusions:**

Changes in the abundances of specific gut microbiota and their metabolites, such as an increased F/B ratio and a decreased abundance of Lactobacillus, as well as the levels of inflammatory indicators and markers of intestinal barrier dysfunction, may play a crucial role in the pathogenesis of BPH. These factors may become effective diagnostic means and potential therapeutic targets for BPH.

**Supplementary Information:**

The online version contains supplementary material available at 10.1186/s12894-025-02003-2.

## Introduction

Benign prostatic hyperplasia (BPH) is one of the common urological diseases in elderly men, caused by disordered proliferation of epithelial and fibromuscular tissue in the transition zone and periurethral glandular regions, leading to prostate enlargement. BPH patients typically present with a series of lower urinary tract symptoms (LUTS) [1]. LUTS caused by BPH significantly reduce men’s quality of life by affecting daily activities and psychological state, although its pathophysiological mechanisms have not yet been fully elucidated [2, 3]. Due to increasing male life expectancy, the prevalence of BPH is projected to continue rising in the near future [4]. Therefore, taking measures to minimize the risk of developing BPH is crucial. While the exact mechanisms of BPH development remain unclear, its etiology is recognized as multifactorial [5]. Chronic inflammation induced by factors such as microbes, bacteria, or viruses can promote pathological changes in the prostate, triggering benign hyperplasia [6].

The gut microbiota (GM) consists of trillions of bacteria, viruses, parasites, and fungi [7]. These microorganisms produce various substances that directly or indirectly affect the physiological functions of host target organs. The GM is a dynamic system that changes according to physical health status and plays a key role in intestinal homeostasis, gut mucosal barrier function, growth and development, metabolism, and immune regulation. It is a critical factor in maintaining the balance between host health and disease [8, 9], Recent research on the impact of changes in GM function and composition on BPH has increased [10], leading to the proposal of the “gut-prostate axis” concept [11]. Alterations in the GM and its derived metabolites can activate the immune system and influence prostate health [12, 13], For instance, butyrate and acetate can inhibit inflammation in mesangial cells induced by high glucose and lipopolysaccharide (LPS), thereby reducing the occurrence of BPH [14].

Gut bacteria such as Eisenbergiella and Ruminococcus (UCG009) appear to have a preventive effect against BPH, whereas Escherichia shigella is positively correlated with an increased risk of BPH development [15]. These findings collectively suggest that the progression of BPH may be associated with GM dysbiosis. These cohort studies and animal experiments provide initial insights into the impact of gut dysbiosis on BPH. However, due to variations in human subject selection and animal models, the research results are inconsistent. Consequently, the specific characteristics of the GM in BPH remain to be fully determined.

Herein, this systematic review seeks to: (1) characterize the differences in gut microbiome composition between patients with BPH and healthy individuals by synthesizing clinical and preclinical evidence; (2) preliminarily explore the key pathways (e.g., systemic inflammation, short-chain fatty acid metabolism, and hormonal regulation) through which GM dysbiosis may contribute to the pathophysiological processes of BPH, based on the available evidence; and (3) summarize the microbial taxa consistently reported to be associated with BPH in both preclinical and clinical studies, thereby proposing potential candidate targets for future mechanistic investigation and targeted therapeutic development along the “gut-prostate axis.”

## Methods

### Search strategy

The protocol for this review was preregistered with PROSPERO(CRD420250632333).We performed a systematic literature review according to the preferred reporting Items for Systematic Reviews and Meta-Analyses (PRISMA) Checklist [16], Three databases were screened, and a systematic electronic literature search was conducted on PubMed, MEDLINE, and Web of Science from the time when these databases were established until October 2025. The following keywords and restrictions were used.: (“Gastrointestinal Microbiome” [Mesh] OR Gastrointestinal Microbiomes) AND ((“Prostatic Hyperplasia“[Mesh]) OR (Hyperplasia, Prostatic))Examples of the complete search terms and search strategies can be found in (Supplementary Table 1).

### Inclusion and exclusion criteria

Inclusion criteria: Clinical/preclinical original studies; Studies on the impact of the GM on BPH; Studies on the correlation between BPH and the composition of the GM; Studies on the intervention of the GM.Exclusion criteria: Review articles; Mendelian study; Studies that do not involve the impact of the GM on BPH; Articles not in the English language.

### Selection of studies

Two reviewers independently screened the titles and abstracts of articles obtained through the search strategy, and then proceeded to the full text for eligible criteria. Any disagreements were resolved through consensus.

### Data extraction and statistical description

Two reviewers carried out data extraction. For preclinical studies, the following data were extracted: authors, animal models, analytical methods, interventions, the differential abundance of GM, and the functional characterization of GM. For clinical studies, the data extracted were authors, regions, demographics (age), sample size, analytical methods, outcome measures (IPSS scores, QoL scores), the differential abundance of GM, and the functional characterization of GM.

## Results

**Fig. 1:**
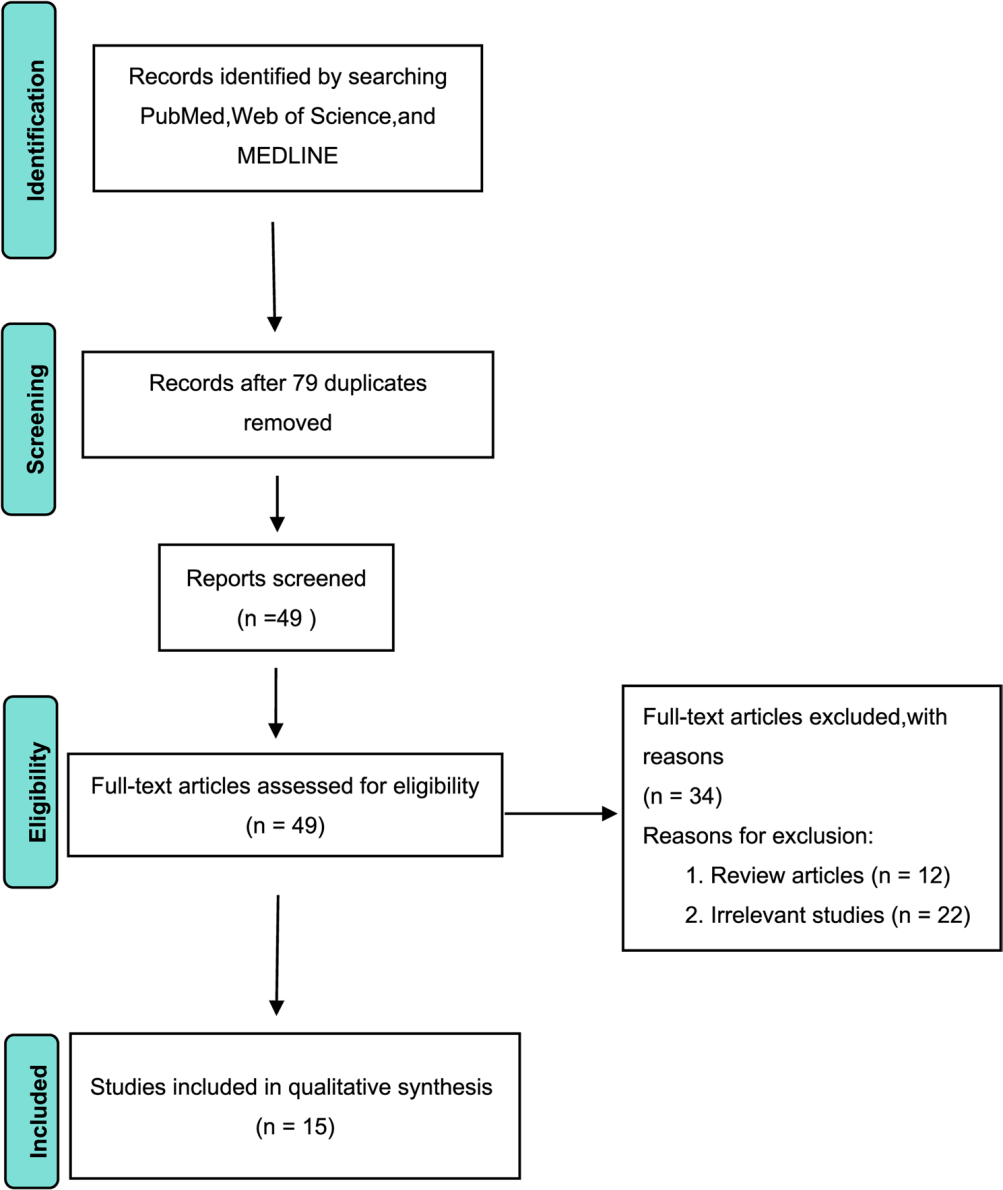
Flow chart of process undertaken to identify eligible studies, according to the PRISMA guidelines

A total of 128 studies were extracted through electronic database search. 15 studies (10 pre-clinical trials [17–26] and 6 clinical trials [25, 27–31], It should be noted that one study [25] contributed to both the preclinical and clinical evidence.) were included in this systematic review (See Fig. 1 for PRISMA flow diagram), These studies included a total of 413 patients with BPH, 338 controls (Table 1), and five different types of BPH mouse models (Table 2). Six preclinical studies [20–26] and four clinical studies [25, 29–31] analyzed the composition of the GM using 16 S rRNA sequencing technology. Two clinical studies [27, 28] used gas chromatography. Next-generation sequencing (NGS) was used in one animal study17, Metagenome Sequencing Analysiswas used in another animal study19.

**Table 1:** Characteristics of clinical studies included in this systemic review

Study	Region	BPH(*n*)	Control (*n*)	Ages	Sequencing	IPSS	QoLs	Higher abundance of gut microbiota	Lower abundance of gut microbiota	Conclusion
Ratajczak W et al.2021[22]	Poland	103	80	BPH:54.00 Hcs:67.00	Gas chromatography	BPH:21.00 Hcs: 3.00	BPH: 3.5 Hcs༚1	isobutyric acid (C4:0i) and isovaleric acid (C5:0i)	N/A	increased levels of isobutyric acid (C4:0i) and isovaleric acid (C5:0i) in the feces of patients with BPH, The gut microbiota is very likely to be indirectly involved in the development of BPH through isobutyric and isovaleric acid
Tsai KY et al.2022[23]	Taiwan	77	46	BPH:69.44 ± 8.23hCS:62,84 ± 7,70	16 S rRNA	BPH:8,74 ± 6,88Hcs: 2.29 ± 2.07	BPH: 2.56 ± 1.21 Hcs༚1.18 ± 0.44	*Alcaligenes*, *Pseudomonas*, *Lactobacillus*, *Akkermansia*, and *Cetobacterium*.	N/A	The top five microbial genera differing significantly between BPH patients and control groups were Alcaligenes, Pseudomonas, Lactobacillus, Akkermansia, and Cetobacterium. Urine microbiota may impact prostate disease development.
Ratajczak W et al.2023[21]	Poland	61	42	BPH:66.25BPH + Mets: 66.76	Gas chromatography	BPH:20.02 BPH with MetS :19.42	BPH:3.19 BPH with MetS:3.44	N/A	N/A	through SCFAs, the gut microbiota can act to prevent or create an inflammatory microenvironment in the prostate gland
Takezawa K et al.2021[24]	Japan	66	62	PE: 70 Non-PE༚71	16 S rRNA	N/A	N/A	F/B	N/A	The F/B ratio of the gut microbiota was associated with prostate enlargement
Wu S et al. [25]	China	30	27	BPH: 68.44 ± 6.38 Control: 50.80 ± 5.67	16 S rRNA	BPH:24.44 ± 4.30 Control: NA	N/A	Bacteroides, Bifidobacterium, Lactobacillus, Ruminococcus_gnavus_group Enterococcus, Streptococcus and Enterobacter	Blautia, Akkermansia, Roseburia, Phascolarctobacterium, Subdoligranulum, and Agathobacter	Akkermansia muciniphila might modulate the gut microbiota-prostate axis and MetS
Ratajczak-Zacharko W[31].	Poland	76	81	BPH: 65.79 Control: 54.66	16 S rRNA	BPH:18.62 Control:5.14	N/A	g__Blautia、g__Bacteroides、g__Faecalibacterium、g__Faecalibacterium、f__Lachnospiraceae、g__Prevotella	N/A	In the cohort of men with BPH, the most prevalent was enterotype 3, characterized by a predominance of Blautia, Bacteroides, and Streptococcus

*Abbreviations*: *BPH *benign prostatic hyperplasia.Mets, metabolic syndrome, *HCs *healthy controls, *IPSS *International Prostate Symptom Score, *QoLs *Quality of Life score, *F/B* *Firmicutes*/*Bacteroidetes, N/A *not available

**Table 2:** Characteristics of animal studies included in this systemic review

Study	Experimental group	Control group	Intervention	Sequencing	Increased abundance of gut microbiota	Decreased abundance of gut microbiota	Conclusion
An J et al.2023 [17]	Male Wistar Hannover rats	Male Wistar Hannover rats	Testosterone undecanoate	NGS	*Ruminococcaceae*,* Flavonifractor*,* Acetatifactor*,* Oscillibacter*,* Pseudoflavonifractor*,* Intestinimonas*,* Butyricimonas*,* and Anaerotruncus*	*Bacilli*,* Lactobacillus*,* Lactobacillaceae*,* Lachnospiracea_incertae_sedis*,* Lactobacillales*,* and Rhodothermaceae*	Lactobacillus, Anaerobacterium, and Desulfovibrio generahave potential as therapeutic agents for the prevention and treatment of BPH or as indicators of post-therapy BPH status.
Gu L et al.2024[19]	Three-week-old male SD rats	Three-week-old male SD rats	HFD-induced	Metagenome Sequencing Analysis	*Firmicutes and Spirochaetes*	N/A	differentially abundant bacteria played a role in the development of pathological alterations in the prostate through the facilitation of inflammatory responses
Gu M et al.2021[20]	Eight weeks old male C57BL/6 mice	Eight weeks old male C57BL/6 mice	HFD (45% kcal fat/17% kcal sucrose) + TP (7.5 mg/kg body weight	16 S rRNA	*Firmicutes*	*Bacteroidetes*	The gut microbiota can reduce the relatively high expressions of ghrelin and ghrelin receptor in the prostate tissue of mice with benign prostatic hyperplasia
Guo X-P et al.2023[21]	male SPF-grade SD rats	male SPF-grade SD rats	testosterone (5 mg/kg)	16 S rRNA	*Tenericutes*, *Mollicutes*, and *RF39*	N/A	alteration of gut microbiota and metabolites is associated with the
Li L-Y et al.2022[23]	male SPF grade SD rats	male SPF grade SD rats	testosterone (5 mg/kg)	16 S rDNA	*Corynebacteriaceae*,* Prevotellaceae*,* Bacteroidales*,* Bacteroidia*,* Bacteroidetes*,* Prevotella*,* Corynebacterium*	*Firmicutes*, *Clostridiales*, *Clostridia*, *Bacilli*, *Turicibacteraceae*, *Turicibacterales*, *Bifidobacteriaceae*, *Bifidobacteriales*, *Coriobacteriales*, *Coriobacteriia*, *Coriobacteriaceae*, *Lachnospiraceae*, *Turicibacter*, *Bilophila*, *Bifidobacterium*, *Adlercreutzia*, and *Coprococcus*.	BPH is associated with alterations of abundance and diversity of gut microbiota and intestinal metabonomics in rats
Han YY et al.2023[22]	male SD rats	male SPF grade SD rats	testosterone propionate	16 S rDNA	*Firmicutes and Actinobacteria*	*Bacteroidetes*,* Verrucomicrobia*,* and Proteobacteria*	medium dose of FRPP was successful in treating BPH by altering hormone levels and gut microbiota in rats with BPH, FRPP was deemed to have significant potential as a plant-based preparationand health supplement for the treatment of BPH.
Yang Y et al.2024 [24]	male, specific pathogen-free, 8-week-old Sprague-Dawley rats	male, specific pathogen-free, 8-week-old Sprague-Dawley rats	TP (5 mg/kg),	16 S rDNA	*Eggerthellaceae*,* Anaerovoracaceae*	*Prevotellaceae_NK3B31_group*,* Dorea*,* Frisingicoccus*	XJP possesses a synergistic anti-BHP effect through multiple components targeting multiple gut microbiota and metabolic pathways
Wu S et al. [25]	Male C57BL/6 mice	Male C57BL/6 mice	TP (10 mg/kg),	16 S rDNA	*Bacteroides*,* Bifidobacterium*,* Lactobacillus*,* Ruminococcus_gnavus_group Enterococcus*,* Streptococcus and Enterobacter*	*Blautia*,* Akkermansia*,* Roseburia*,* Phascolarctobacterium*,* Subdoligranulum*,* and Agathobacter*	Akkermansia muciniphila might modulate the gut microbiota-prostate axis and MetS
Yang T et al.[26]	Six to eight-week‐old male db/db C57BL/6 diabetes mice	Six to eight-week‐old male db/db C57BL/6 diabetes mice	TP (7.5 mg/kg body weight + 15 mg/kg/day EGCG intragastrically once a day for 4 weeks	16 S rRNA	*Firmicutes*	*Proteobacteria and Saccharibacteria*	EGCG treatment effectively rebalanced gut microbiota composition by reducing Firmicutes dominance and normalizing the Firmicutes/Bacteroidetes ratio

*Abbreviations*: *BPH *benign prostatic hyperplasia, *NGS *Next Generation Sequencing, *TP *testosterone, *SD *Sprague Dawley, *SPF *Specific - Pathogen - Free, *HFD *high-fat diet, *FRPP *Fermented rape pollen powder, *XJP *Xiaojin Pill

### Quality of included studies

After evaluating the six clinical studies using the Newcastle–Ottawa scale [32], two studies scored 8 points (2/6), three studies scored 6 points (3/6), and one study scored 5 points (1/6), indicating that the overall quality of the included studies was generally high (Supplementary Table 2).

### Association between microbiota diversity and BPH

Regarding α-diversity, the findings were inconsistent. One clinical study reported a significant reduction in α-diversity in BPH patients; however, preclinical studies showed divergent results, including decreased, unchanged, or even increased α-diversity (as assessed by indices such as Chao1, Shannon, and Simpson; Supplementary Table 3) [21, 22, 24, 25, 31]. This inconsistency may be attributed to several factors: (1) heterogeneity among study populations, (2) differences in disease stages or experimental models, and (3) methodological variations. In contrast, the majority of studies (five preclinical and three clinical) (references) observed significant differences in β-diversity between BPH and control groups, indicating that structural alterations in the gut microbial community represent a more consistent finding in the context of BPH [17, 19, 21–25, 30, 31].

### Core microbiota changes in BPH

At the phylum level, the BPH group exhibited a higher Firmicutes/Bacteroidetes (F/B) ratio compared to the control group, a finding consistently confirmed in both animal studies [19, 20, 22–24, 30] and clinical research.At the genus level, animal studies [17, 22–24] consistently reported a lower abundance of Lactobacillus in the BPH group. However, clinical findings were heterogeneous: one study [29] supported this observation, while another [25] reported an upregulation of this genus in the BPH group. Furthermore, multiple animal studies [17, 23, 24] and clinical studies [25, 31] jointly identified a higher abundance of Prevotellaceae in the BPH group. Both animal [17, 19, 21–23] and clinical [25] studies also revealed an increased abundance of Ruminococcus in the BPH group. Additional animal studies [22, 23] reported changes in the abundance of Turicibacter and Clostridium. An independent clinical study further indicated that an enterotype dominated by Blautia, Bacteroides, and Streptococcus was associated with an increased risk of BPH [31], potentially representing a specific pathogenic microbial pattern. In summary, despite some discrepancies at the genus level, animal and clinical studies demonstrate significant consistency in the core microbiota alterations associated with BPH. These repeatedly validated microbial features enhance the potential of GM as non-invasive biomarkers for BPH risk or progression.

### Potential mechanistic links between gut dysbiosis and BPH pathophysiology

Two clinical [25, 27] studies and four animal studies [17, 20, 23, 24] reported changes in inflammatory markers and apoptosis markers.The abundance of Lactobacillus was associated with promoting prostate apoptosis progression, whereas the abundance of Acetatifactor and Butyricimonas was associated with inhibiting prostate apoptosis [17]. Gut microbiota-associated ghrelin was also found to inhibit prostate apoptosis [20]. The inflammatory marker interleukin-6 (IL-6) is elevated in patients with BPH but can be reduced through intervention with specific probiotic strains, such as Bifidobacterium longum BLG1 and Bifidobacterium psychraerophilum Q5 [18]. Similarly, Akkermansia muciniphila has been shown to decrease pro-inflammatory cytokines including TNF-α, IL-1, and IL-6, while promoting an increase in the anti-inflammatory cytokine IL-10 [25]. Further supporting the link between microbial metabolites and inflammation, IL-6 levels are negatively correlated with hexanoate and butyrate, but positively correlated with acetate. Concurrently, IL-18 exhibits a moderate positive correlation with isocaproate [27]. This suggests that alterations in the short-chain fatty acid (SCFA) profile may mediate the microbiota’s effect on prostate inflammation.

### Causal validation of microbiota function and metabolic pathways in BPH

Animal studies based on KEGG analysis revealed significantly enriched pathways in BPH models, closely related to the core pathophysiological mechanisms of BPH: (1) Steroid hormone biosynthesis pathway enrichment. (2) Unsaturated fatty acid biosynthesis pathway enrichment [21–23]. (3) Pyrimidine metabolism (1 study) [24], NLR signaling pathway, estrogen signaling pathway, and the key PI3K-Akt signaling pathway (1 study) enrichment [19]. Gut dysbiosis may collectively contribute to the development and progression of BPH by influencing SCFA production (e.g., butyrate, affecting inflammation and apoptosis), steroid hormone metabolism, and activating key signaling pathways (such as PI3K-Akt) that promote cell proliferation and inhibit apoptosis.

## Discussion

**Fig. 2:**
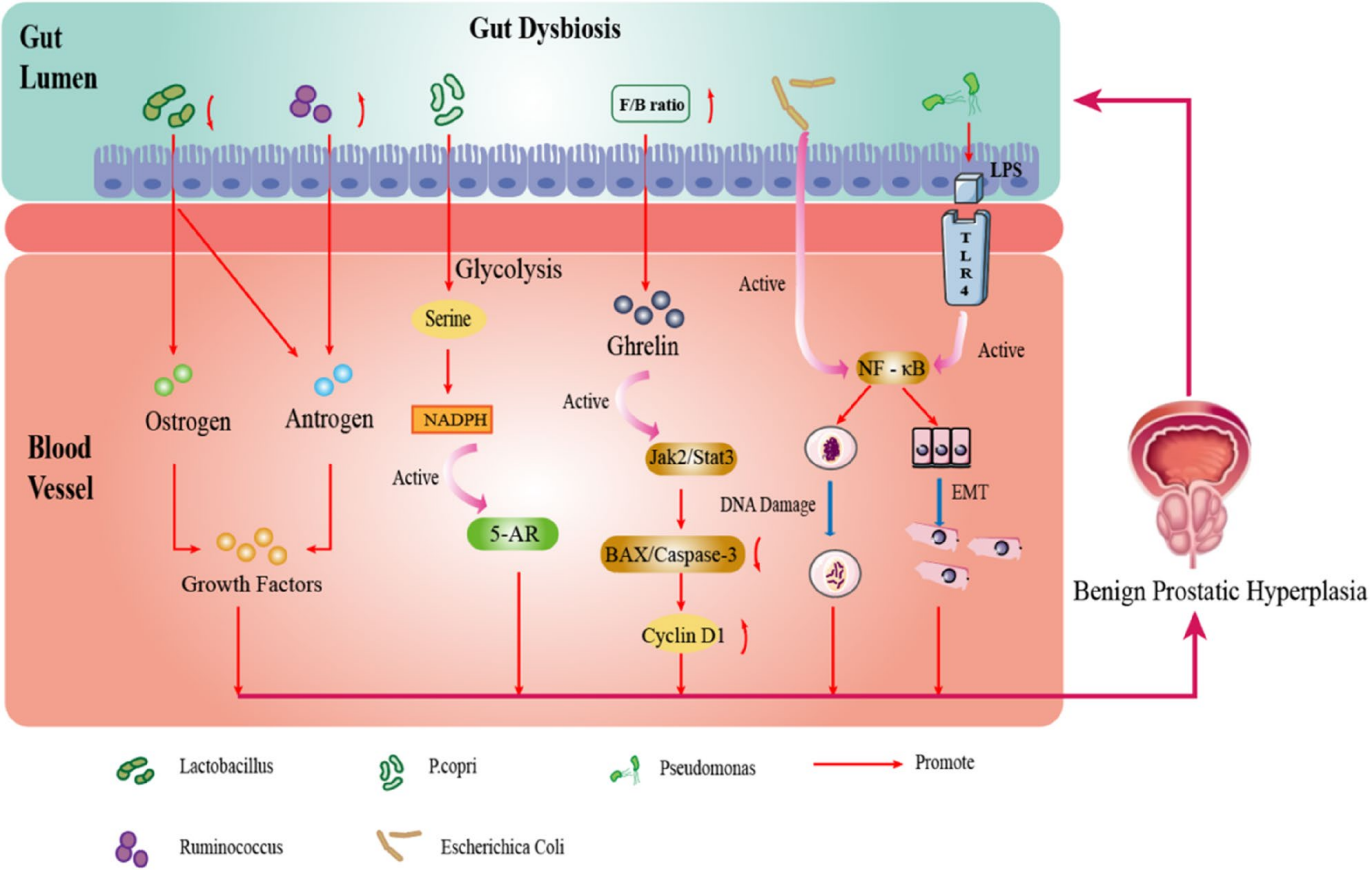
Possible mechanism linking gut microbiota dysbiosis to BPH，Alteration of specific microbial taxa may contribute to the pathogenesis and progression of BPH through the following four factors，First, Lactobacillus and Ruminococcus influence the occurrence and development of BPH by promoting growth factors through hormone metabolism. Second, P.copri may upregulate the activity of 5AR by inducing an increase in NADPH production, thus affecting the progression of BPH. Third, an increase in the F/B may significantly increase the level of ghrelin in prostate tissue, thereby activating the Jak2/Stat3 pathway and suppressing the BAX/Caspase-3 apoptosis pathway and upregulating Cyclin D1, further exacerbating the progression of BPH. Fourth, specific microbiota can trigger DNA damage and the epithelial - mesenchymal transition process in prostate epithelial cells by activating the NF - κB signal transduction, thus exacerbating prostate hyperplasia. BPH, benign prostatic hyperplasia. P.copri, Prevotella copri. 5AR, 5-alpha reductase. NADPH, nicotinamide adenine dinucleotide phosphate.F/B,*Firmicutes*/*Bacteroidetes.*NF- κB, nuclear factor kappa - light - chain - enhancer of activated B cells

This review represents the first systematic exploration of the potential link between GM dysbiosis and the pathogenesis of BPH. Through comprehensive analysis of existing evidence, we have revealed significant alterations in the gut microbiome characteristics of BPH and preliminarily outlined a core mechanistic framework through which microbiota may influence prostatic homeostasis via multiple pathways including metabolism, inflammation, and barrier function. Our analysis demonstrates that the gut microbiome structure of BPH differs significantly from that of healthy individuals (as indicated by PCoA), primarily manifesting as changes in community composition rather than a mere reduction in within-individual α-diversity (as suggested by Chao1/Simpson indices). Specifically, an increased F/B ratio, elevated abundance of Prevotella (particularly P. copri) and Ruminococcus, along with decreased Lactobacillus abundance(Fig. 2), collectively form the core microbial signature closely associated with BPH. These changes do not occur in isolation but rather interact synergistically, ultimately promoting prostatic hyperplasia.

### The elevation of the F/B ratio plays a significant role in the pathogenesis of BPH

The Firmicutes and Bacteroidetes phyla represent the two dominant bacterial groups in the mammalian gut, and alterations in their ratio (F/B) have been recognized as a potential biomarker for various pathological conditions [33, 34]. In animal models, an elevated F/B ratio promotes the secretion of ghrelin, which activates the Jak2/Stat3 signaling pathway in both prostate stromal and epithelial cells. This activation directly stimulates abnormal cell proliferation and survival, accompanied by suppression of the BAX/Caspase-3 apoptosis pathway and upregulation of pro-proliferative genes such as Cyclin D1, thereby driving the development of BPH [20, 35, 36]. Studies have further demonstrated that restoring a dysregulated F/B ratio can attenuate BPH progression [26]. Clinically, a significantly higher F/B ratio has been observed in patients with a prostate volume >30 mL, suggesting its potential value as a non-invasive diagnostic biomarker for BPH [30]. In summary, evidence from both mechanistic studies in animals and clinical observations supports an important role of the GM F/B ratio in the pathophysiology of BPH. However, as key human evidence remains limited to cross-sectional studies, a causal relationship warrants further validation through prospective investigations.

### An augmented presence of prevotella copri might be a characteristic indicator exclusive to BPH

The increased abundance of Prevotella copri (P. copri) in the GM may be an important part of the pathogenesis of BPH. P. copri can participate in the development of BPH by regulating inflammation or metabolism [37]. P. copri is involved in various processes of carbohydrate metabolism in the body, such as glycolysis, gluconeogenesis, and glucose transport. In the glycolysis process, P. copri enhances serine synthesis via the phosphoserine aminotransferase 1 (PSAT1) and phosphoserine phosphatase (PSPH) pathway [38]. Serine serves as a key precursor for cellular production of nicotinamide adenine dinucleotide phosphate (NADPH, reduced form) – an essential cofactor required to activate prostatic 5α-reductase (5AR, particularly the 5AR2 isoform) [39]. The 5AR enzyme catalyzes the conversion of testosterone to dihydrotestosterone (DHT), the primary androgen driving BPH progression [40, 41]. Thus, P. copri may accelerate BPH pathogenesis through a “glycolysis-PSAT1-PSPH axis” that promotes serine synthesis, boosts NADPH generation, and consequently enhances 5AR-mediated DHT production from testosterone.These findings reveal a novel molecular mechanism by which GM remotely regulates local androgen metabolism in the prostate, providing fresh insights into the gut-prostate axis in BPH development.

### Ruminococcus plays a distinct role in BPH pathogenesis

Ruminococcus is an anaerobic, Gram-positive genus within the Lachnospiraceae family of the Firmicutes phylum [42], known for its strong substrate-degrading capacity and association with various inflammatory diseases [43]. This genus is notable for producing butyrate, which helps maintain gut health, enhance intestinal function, and reduce inflammatory cytokine levels [44], Importantly, Ruminococcus is also capable of metabolizing androgens, and its abundance correlates positively with serum testosterone levels [45]. Sex hormone imbalance is one of the most important mechanisms in the occurrence and development of BPH, and the abnormal production and metabolism of androgens, mainly testosterone, is the most critical factor in this mechanism [46]. In BPH models, a reduction in Ruminococcus may impair the gut’s ability to metabolically clear androgens, leading to a relative increase in systemic testosterone. This hormonal shift subsequently stimulates the expression of growth factors such as IGF-1 and EGF, ultimately exacerbating hyperplastic changes in prostate tissue [45, 47]. Thus, decreased abundance of Ruminococcus may contribute to BPH pathogenesis through multiple mechanisms—including impaired intestinal barrier function, reduced butyrate production, and dysregulated androgen metabolism—though the precise pathways involved require further investigation.

### The complex role of lactobacillus in BPH pathogenesis

Lactobacillus is a genus comprising approximately 310 species, widely present in dairy products and renowned for its probiotic properties [48], Certain strains within this genus help the host resist pathogens and exert therapeutic effects in conditions such as gastrointestinal disorders, vaginal infections, and dermatological diseases like eczema [49, 50]. The role of Lactobacillus in BPH is complex. Although most studies report a decreased abundance of Lactobacillus in the gut, one study found a positive correlation between urinary Lactobacillus and prostate-specific antigen (PSA) levels [51], This discrepancy may stem from ecological niche differences (gut vs. urinary tract) and strain-specific functions: a reduction in intestinal Lactobacillus may indicate a loss of protective homeostasis, whereas the proliferation of specific strains in the urinary tract could act as opportunistic pathogens and influence PSA levels through pro-inflammatory mechanisms—PSA itself being a recognized risk marker for BPH [52]. On the other hand, some Lactobacillus strains are capable of metabolizing estrogen [53], Estrogen can induce the expression of lipopolysaccharide-binding protein (LBP) in prostate stromal cells, potentially enhancing the sensitivity of prostate cells to LPS and thereby amplifying inflammatory cascades [54]. In a BPH rat model, reduced levels of Lactobacillus led to elevated systemic estrogen, which subsequently promoted the overexpression of growth factors such as insulin-like growth factor (IGF) and epidermal growth factor (EGF). This stimulated the proliferation of both epithelial and stromal cells, accelerating the progression of prostatic hyperplasia [22]. Notably, after treatment with finasteride, the relative abundance of Lactobacillus in the gut of BPH rats increased significantly and showed a negative correlation with levels of dihydrotestosterone (DHT) and 5α-reductase [17]. Although the therapeutic effect is primarily attributed to the drug itself, the GM may participate in modulating the treatment response: the restoration of Lactobacillus could be a consequence of the drug’s action or may synergize with the medication through yet-uncharacterized pathways. In summary, Lactobacillus may influence the progression of BPH through multiple mechanisms, including modulation of hormone metabolism and inflammatory responses, highlighting its potential as a therapeutic target.

### The microbiota-inflammation-barrier axis and pelvic neural cross-sensitization synergistically drive the pathogenesis of BPH

GM interacts with the host to modulate both local and systemic immune and inflammatory responses, in which the function of the intestinal barrier plays a crucial role [55]. The intestinal barrier, composed of the mucus layer, the intestinal epithelial layer, and the inner layer, serves as the core component of the intestinal immune system [56]. GM maintains the integrity of the intestinal barrier through multiple mechanisms: the SCFAs it produces enhance tight junctions, promote mucus and IgA secretion, increase transepithelial electrical resistance (TEER), and inhibit LPS-induced NLRP3 inflammasome activation and autophagy [57, 58]; meanwhile, GM also regulates the secretion of glucagon-like peptide-2 (GLP-2). GLP-2 expression positively correlates with zonula occludens-1 (ZO-1) and enhances barrier function by promoting crypt cell proliferation, reducing epithelial apoptosis, and decreasing intestinal permeability, thereby alleviating systemic inflammation and oxidative stress; ZO-1, in turn, acts as a structural scaffold, bridging claudins, occludin, and the cytoskeleton to maintain the stability of the tight junction complex [59, 60]. However, gut dysbiosis (e.g., a reduction in SCFA-producing bacteria such as Faecalibacterium prausnitzii) disrupts these protective mechanisms, leading to impaired intestinal barrier function. This not only facilitates the translocation of gut bacteria and their metabolites (including SCFAs and toxins such as LPS) into the bloodstream—where LPS can exacerbate inflammation by activating the IL-6–STAT3 signaling axis—but dysbiosis itself can also trigger the release of pro-inflammatory cytokines such as IL-17 and TNF-α, collectively driving systemic immune-inflammatory responses. These circulating harmful substances and inflammatory factors can reach the prostate and contribute to a local inflammatory microenvironment by activating signaling pathways such as NF-κB [28, 61, 62]. For example, Escherichia coli detected in BPH tissues can activate the NF-κB pathway, induce DNA damage in prostate epithelial cells, and lead to abnormal proliferation or apoptosis [63]. Pseudomonas species enriched in BPH tissues derive LPS that activates NF-κB via TLR4, promoting the release of IL-6/IL-18, enhancing cell proliferation, inducing epithelial-mesenchymal transition, and inhibiting apoptosis, thereby driving prostate tissue hyperplasia [64–66]. Furthermore, “cross-sensitization” exists between the gut and the prostate: afferent signals from the lower urinary tract and the distal intestine can interact via central and peripheral neural mechanisms, leading to abnormal sensory associations between the two organs. The underlying mechanisms may involve direct sensitization of afferent nerve endings, enhanced signaling in dorsal root ganglia and spinal pathways, and indirect peripheral nerve sensitization triggered by epithelial barrier disruption and neurogenic inflammation. Such neuroplastic changes can lead to a state of neuronal hypersensitivity. Modulating the GM may intervene in these shared neural pathways, offering new strategies for alleviating prostate-related LUTS [67, 68]. In summary, gut dysbiosis may play a key pathogenic role in the development of BPH by impairing barrier function, promoting systemic inflammation and immune dysregulation, and facilitating cross-sensitization phenomena.

### Potential impact of microbial metabolites on BPH development mechanisms

Metabolites of the GM serve as crucial mediators in host-microbiota crosstalk, primarily including SCFAs, trimethylamine N-oxide, branched-chain amino acids, bile acids, and more [69], Among these, SCFAs—comprising acetate, propionate, and butyrate—represent one of the most significant metabolite groups derived from GM [70]. with demonstrated roles in modulating immune responses, energy metabolism, and cell proliferation [71, 72]. In terms of metabolic regulation, butyrate and propionate influence gluconeogenesis and promote satiety via vagal nerve signaling [73–75], Butyrate further reduces energy intake through gut-brain neural circuits while enhancing fat oxidation via activation of brown adipose tissue [74], Acetate, upon crossing the blood-brain barrier, also contributes to hypothalamic satiety signaling [76], These mechanisms suggest that SCFAs may indirectly influence BPH risk by ameliorating obesity—a well-established risk factor for BPH. Notably, elevated levels of isobutyrate (C4:0i) and isovalerate (C5:0i) have been observed in BPH patients, implying a potential link between branched-chain SCFAs (such as isohexanoate, C6:0i) and susceptibility to BPH [28]. Conversely, gut dysbiosis—often characterized by reduced SCFA-producing bacteria and expansion of potentially pathogenic species—can lead to systemic dissemination of pro-inflammatory cytokines (e.g., IL-17, IL-23, TNF-α, and IFN-γ), which may reach the prostate and provoke local inflammation [13]. Current evidence indicates that SCFAs are likely involved in the pathophysiology of BPH. However, observational studies to date cannot establish a causal relationship between SCFAs and BPH progression. Further elucidation of the complex interplay between gut microbial metabolites and BPH may open new avenues for future intervention strategies.

### The microbiota-based therapeutic prospects of BPH

**Fig. 3:**
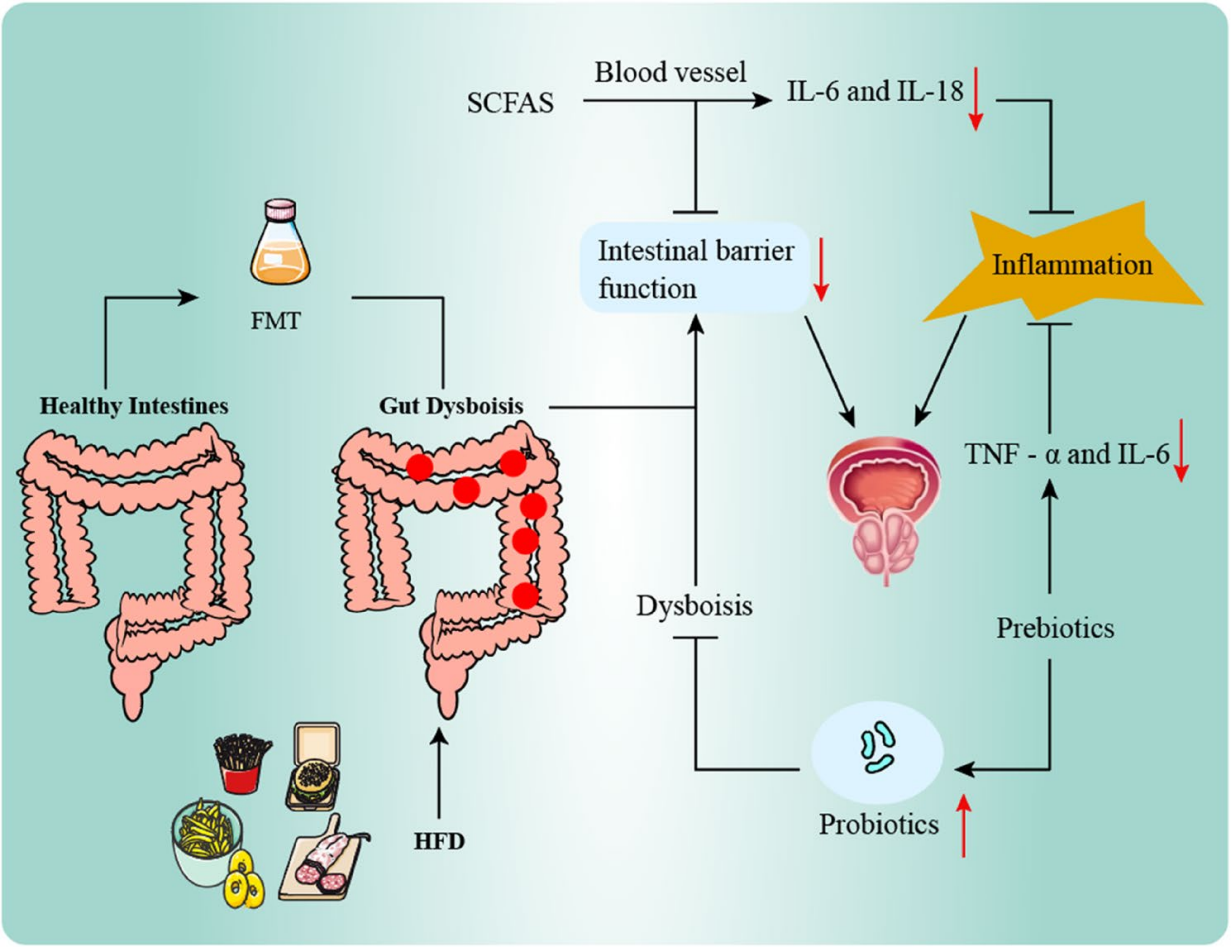
HFD can lead to an imbalance in gut bacteria and their metabolites, which in turn causes intestinal leakage. FMT is the transplantation of gut microbiota from healthy donors into patients to regulate the imbalance of gut flora.Prebiotics can significantly reduce the levels of TNF - α and IL - 6 in the serum of mice, alleviating systemic inflammation. On the other hand, probiotics are fermented by beneficial bacteria in the gut and stimulate the growth of probiotics in the intestine. Supplementation with probiotics can regulate the composition of the gut microbiota to correct the imbalance of gut flora.SCFAS supplements can remarkably alter the composition of the gut microbiota, protect the function of the intestinal barrier, and reduce the levels of IL - 6 and IL - 18 in the ileum to inhibit the inflammatory response.Ultimately, these can relieve the occurrence and development of benign prostatic hyperplasia. HFD, high - fat diet, FMT, fecal microbial transplant; SCFAs, short-chain fatty acids; IL, interleukin; TNF-α, tumor necrosis factor-α

Modulating the GM offers a novel perspective for intervening in BPH. Explorations have been conducted in areas such as diet, SCFAs, probiotics, prebiotics, and fecal microbiota transplantation (FMT). Although related research remains predominantly in the pre-clinical stage, these efforts have laid a significant foundation for understanding the mechanisms of the “gut-prostate axis” (Fig. 3). In terms of nutritional interventions, animal studies indicate that obesity and a high-fat diet can disrupt gut homeostasis and exacerbate BPH, whereas a Mediterranean dietary pattern (increased intake of fruits and vegetables, reduced red meat consumption) demonstrates potential ameliorative effects. Natural active compounds, such as the green tea polyphenol EGCG, have been shown in mouse models to alleviate BPH progression by inhibiting the IGF-1/PI3K/AKT signaling pathway [20, 77, 78]. Furthermore, in the field of probiotics, pre-clinical studies suggest that supplementation with Bifidobacterium longum, Bifidobacterium psychraerophilum, or Akkermansia muciniphila may enhance the expression of intestinal tight junction proteins (such as ZO-1 and claudin-1), improve barrier function, and modulate metabolic parameters related to BPH [18, 25, 79]. Additionally, prebiotics such as indigestible components like polyphenols and oligosaccharides exhibit potential in animal models for improving intestinal inflammation and metabolic disorders, leveraging their antioxidant, anti-inflammatory properties, and microbiota-modulating functions [80, 81]. For instance, grape polyphenols, after metabolism by gut bacteria, can reduce levels of TNF-α, IL-6, and LPS, suggesting a potential indirect influence on BPH development by mitigating leaky gut and associated systemic inflammation. However, this effect requires clinical validation [82]. Finally, FMT representing a more fundamental interventional approach, has demonstrated potential in animal studies to remotely influence the prostate by remodelling the GM [83], Its mechanisms may involve two aspects: firstly, increasing the production of beneficial metabolites like butyrate, which accumulates in prostate tissue and can inhibit proliferation and induce apoptosis; and secondly, upregulating the expression of G protein-coupled estrogen receptor in prostate tissue, thereby suppressing the hyperplastic process [84].Although FMT provides a new perspective for BPH treatment, it is currently still in the experimental exploration stage, and its safety and efficacy require systematic evaluation before clinical application can be considered.

This study aims to provide evidence supporting the potential mechanistic links between GM dysbiosis and the pathogenesis of BPH, while offering insights for novel preventive and therapeutic strategies. However, several limitations should be acknowledged. The limited number of clinical studies and substantial methodological heterogeneity precluded meta-analysis and weakened our ability to draw definitive conclusions. More importantly, the predominance of cross-sectional observational designs impedes causal inference between GM alterations and BPH. In mechanistic exploration, animal models (e.g., testosterone-induced) only partially recapitulate human BPH pathophysiology, limiting the translational relevance of preclinical findings. Technically, the predominant reliance on 16 S rRNA sequencing restricted our capacity to characterize microbial changes at species/strain resolution and functional levels. Furthermore, observational studies are inherently confounded by factors such as age and BMI. While restricting our literature search to English publications may introduce language bias, we note that core research in this field is predominantly published in English, and thus this limitation likely has minimal impact on our primary conclusions. Future research should prioritize well-designed mechanistic investigations and rigorously controlled clinical trials to: (1) establish causality, (2) elucidate underlying molecular pathways, and (3) validate therapeutic interventions. Such advances will facilitate the development of microbiota-targeted management strategies for BPH.

## Conclusion

This systematic review found among the 15 included studies that an increased F/B ratio might be a specific marker of the GM in BPH, which is associated with increased ghrelin secretion and prostate hypertrophy. Besides, the increased abundance of Prevotella might also be a specific marker of the GM in BPH, and it might influence the occurrence and development of BPH by participating in the processes of inflammation and glucose metabolism. In addition, the enrichment of Lactobacillus and Ruminococcus might also play a role in the pathogenesis of BPH by regulating hormone metabolism. These findings contribute to a deeper understanding of the impact of the GM on BPH and its role in the prevention and treatment of BPH.

Flow chart of process undertaken to identify eligible studies, according to the PRISMA guidelines.

Possible mechanism linking gut microbiota dysbiosis to BPH, Alteration of specific microbial taxa may contribute to the pathogenesis and progression of BPH through the following four factors, First, Lactobacillus and Ruminococcus influence the occurrence and development of BPH by promoting growth factors through hormone metabolism. Second, P.copri may upregulate the activity of 5AR by inducing an increase in NADPH production, thus affecting the progression of BPH. Third, an increase in the F/B may significantly increase the level of ghrelin in prostate tissue, thereby activating the Jak2/Stat3 pathway and suppressing the BAX/Caspase-3 apoptosis pathway and upregulating Cyclin D1, further exacerbating the progression of BPH. Fourth, specific microbiota can trigger DNA damage and the epithelial - mesenchymal transition process in prostate epithelial cells by activating the NF - κB signal transduction, thus exacerbating prostate hyperplasia. BPH, benign prostatic hyperplasia. P.copri, Prevotella copri. 5AR, 5-alpha reductase. NADPH, nicotinamide adenine dinucleotide phosphate.F/B, *Firmicutes*/*Bacteroidetes.* NF - κB, nuclear factor kappa - light - chain - enhancer of activated B cells.

HFD can lead to an imbalance in gut bacteria and their metabolites, which in turn causes intestinal leakage. FMT is the transplantation of gut microbiota from healthy donors into patients to regulate the imbalance of gut flora.Prebiotics can significantly reduce the levels of TNF - α and IL − 6 in the serum of mice, alleviating systemic inflammation. On the other hand, probiotics are fermented by beneficial bacteria in the gut and stimulate the growth of probiotics in the intestine. Supplementation with probiotics can regulate the composition of the gut microbiota to correct the imbalance of gut flora. SCFAS supplements can remarkably alter the composition of the gut microbiota, protect the function of the intestinal barrier, and reduce the levels of IL − 6 and IL − 18 in the ileum to inhibit the inflammatory response.Ultimately, these can relieve the occurrence and development of benign prostatic hyperplasia. HFD, high - fat diet, FMT, fecal microbial transplant; SCFAs, short-chain fatty acids; IL, interleukin; TNF-α, tumor necrosis factor-α.

## Supplementary Information

Supplementary Material 1.

## Data Availability

All data generated or analyzed in the course of this study are included in the document and its supplementary materials. Further inquiry can be available from the corresponding author.
