# Supplementary material for: The gut-prostate axis in benign prostatic hyperplasia: systematic review of microbial dysbiosis and pathogenic mechanisms
Source: BMC Urol. 2026 Feb 2;26:26. doi: 10.1186/s12894-025-02003-2 (PMC12866195; doi:10.1186/s12894-025-02003-2)
Supplement: Supplementary file 1 — Supplementary Material 1. [file 12894_2025_2003_MOESM1_ESM.docx]

***Supplementary Material***

Supplementary table 1 Search strategy

Supplementary table 2 Quality assessment of selected studies using the Newcastle-Ottawa Scale

Supplementary table 3 Alpha diversity and Beta diversity between BPH and Control

Supplementary table 1 Search strategy

PubMed

Search number Query Results

| ID | Search | Results |
| --- | --- | --- |
| 15 | ("Gastrointestinal Microbiome"[Mesh] OR Gastrointestinal Microbiomes OR Microbiome, Gastrointestinal OR Gastrointestinal Microbial Community OR Gastrointestinal Microbial Communities OR Microbial Community, Gastrointestinal OR Gut Microbiome OR Gut Microbiomes OR Microbiome, Gut OR Gut Microflora OR Microflora, Gut OR Gastrointestinal Microflora OR Microflora, Gastrointestinal OR Gastrointestinal Flora OR Flora, Gastrointestinal OR Gut Flora OR Flora, Gut OR Gastrointestinal Microbiota OR Gastrointestinal Microbiotas OR Microbiota, Gastrointestinal OR Gut Microbiota OR Gut Microbiotas OR Microbiota, Gut OR Intestinal Microbiome OR Intestinal Microbiomes OR Microbiome, Intestinal OR Intestinal Flora OR Flora, Intestinal OR Intestinal Microbiota OR Intestinal Microbiotas OR Microbiota, Intestinal OR Intestinal Microflora OR Microflora, Intestinal OR Enteric Bacteria OR Bacteria, Enteric OR Gastric Microbiome OR Gastric Microbiomes OR Microbiome, Gastric) AND (("Prostatic Hyperplasia"[Mesh]) OR (Hyperplasia, Prostatic or Adenoma, Prostatic or Adenomas, Prostatic or Prostatic Adenomas or Prostatic Adenoma or Prostatic Hypertrophy or Hypertrophies, Prostatic or Hypertrophy, Prostatic or Prostatic Hypertrophies or Benign Prostatic Hyperplasia or Benign Prostatic Hyperplasias or Hyperplasia, Benign Prostatic or Hyperplasias, Benign Prostatic or Prostatic Hyperplasias, Benign or Prostatic Hyperplasia, Benign or Prostatic Hypertrophy, Benign or Benign Prostatic Hypertrophy or Hypertrophy, Benign Prostatic)) | 49 |
| 14 | "Gastrointestinal Microbiome"[Mesh] OR Gastrointestinal Microbiomes OR Microbiome, Gastrointestinal OR Gastrointestinal Microbial Community OR Gastrointestinal Microbial Communities OR Microbial Community, Gastrointestinal OR Gut Microbiome OR Gut Microbiomes OR Microbiome, Gut OR Gut Microflora OR Microflora, Gut OR Gastrointestinal Microflora OR Microflora, Gastrointestinal OR Gastrointestinal Flora OR Flora, Gastrointestinal OR Gut Flora OR Flora, Gut OR Gastrointestinal Microbiota OR Gastrointestinal Microbiotas OR Microbiota, Gastrointestinal OR Gut Microbiota OR Gut Microbiotas OR Microbiota, Gut OR Intestinal Microbiome OR Intestinal Microbiomes OR Microbiome, Intestinal OR Intestinal Flora OR Flora, Intestinal OR Intestinal Microbiota OR Intestinal Microbiotas OR Microbiota, Intestinal OR Intestinal Microflora OR Microflora, Intestinal OR Enteric Bacteria OR Bacteria, Enteric OR Gastric Microbiome OR Gastric Microbiomes OR Microbiome, Gastric | 147478 |
| 13 | ("Prostatic Hyperplasia"[Mesh]) OR (Hyperplasia, Prostatic or Adenoma, Prostatic or Adenomas, Prostatic or Prostatic Adenomas or Prostatic Adenoma or Prostatic Hypertrophy or Hypertrophies, Prostatic or Hypertrophy, Prostatic or Prostatic Hypertrophies or Benign Prostatic Hyperplasia or Benign Prostatic Hyperplasias or Hyperplasia, Benign Prostatic or Hyperplasias, Benign Prostatic or Prostatic Hyperplasias, Benign or Prostatic Hyperplasia, Benign or Prostatic Hypertrophy, Benign or Benign Prostatic Hypertrophy or Hypertrophy, Benign Prostatic) | 34869 |

MEDLINE

| ID | Search | Results |
| --- | --- | --- |
| 1 | Gastrointestinal Microbiome/ | 60029 |
| 2 | Prostatic Hyperplasia/ | 25525 |
| 3 | (Hyperplasia, Prostatic or Adenoma, Prostatic or Adenomas, Prostatic or Prostatic Adenomas or Prostatic Adenoma or Prostatic Hypertrophy or Hypertrophies, Prostatic or Hypertrophy, Prostatic or Prostatic Hypertrophies or Benign Prostatic Hyperplasia or Benign Prostatic Hyperplasias or Hyperplasia, Benign Prostatic or Hyperplasias, Benign Prostatic or Prostatic Hyperplasias, Benign or Prostatic Hyperplasia, Benign or Prostatic Hypertrophy, Benign or Benign Prostatic Hypertrophy or Hypertrophy, Benign Prostatic).mp. [mp=title, book title, abstract, original title, name of substance word, subject heading word, floating sub-heading word, keyword heading word, organism supplementary concept word, protocol supplementary concept word, rare disease supplementary concept word, unique identifier, synonyms, population supplementary concept word, anatomy supplementary concept word] | 21717 |
| 4 | (Gastrointestinal Microbiomes or Microbiome, Gastrointestinal or Gastrointestinal Microbial Community or Gastrointestinal Microbial Communities or Microbial Community, Gastrointestinal or Gut Microbiome or Gut Microbiomes or Microbiome, Gut or Gut Microflora or Microflora, Gut or Gastrointestinal Microflora or Microflora, Gastrointestinal or Gastrointestinal Flora or Flora, Gastrointestinal or Gut Flora or Flora, Gut or Gastrointestinal Microbiota or Gastrointestinal Microbiotas or Microbiota, Gastrointestinal or Gut Microbiota or Gut Microbiotas or Microbiota, Gut or Intestinal Microbiome or Intestinal Microbiomes or Microbiome, Intestinal or Intestinal Flora or Flora, Intestinal or Intestinal Microbiota or Intestinal Microbiotas or Microbiota, Intestinal or Intestinal Microflora or Microflora, Intestinal or Enteric Bacteria or Bacteria, Enteric or Gastric Microbiome or Gastric Microbiomes or Microbiome, Gastric).mp. [mp=title, book title, abstract, original title, name of substance word, subject heading word, floating sub-heading word, keyword heading word, organism supplementary concept word, protocol supplementary concept word, rare disease supplementary concept word, unique identifier, synonyms, population supplementary concept word, anatomy supplementary concept word] | 110591 |
| 5 | 1 or 4 | 121227 |
| 6 | 2 or 3 | 32392 |
| 7 | 5 and 6 | 40 |

Web of Science

| ID | Search Query | Results |
| --- | --- | --- |
| 1 | Prostatic Hyperplasia (Topic) | 9551 |
| 2 | TS=(Hyperplasia, Prostatic OR Adenoma, Prostatic OR Adenomas, Prostatic OR Prostatic Adenomas OR Prostatic Adenoma OR Prostatic Hypertrophy OR Hypertrophies, Prostatic OR Hypertrophy, Prostatic OR Prostatic Hypertrophies OR Benign Prostatic Hyperplasia OR Benign Prostatic Hyperplasias OR Hyperplasia, Benign Prostatic OR Hyperplasias, Benign Prostatic OR Prostatic Hyperplasias, Benign OR Prostatic Hyperplasia, Benign OR Prostatic Hypertrophy, Benign OR Benign Prostatic Hypertrophy OR Hypertrophy, Benign Prostatic) | 9818 |
| 3 | Gastrointestinal Microbiome (Topic) | 8831 |
| 4 | TS=（Gastrointestinal Microbiomes OR Microbiome, Gastrointestinal OR Gastrointestinal Microbial Community OR Gastrointestinal Microbial Communities OR Microbial Community, Gastrointestinal OR Gut Microbiome OR Gut Microbiomes OR Microbiome, Gut OR Gut MicroflORa OR MicroflORa, Gut OR Gastrointestinal MicroflORa OR MicroflORa, Gastrointestinal OR Gastrointestinal FlORa OR FlORa, Gastrointestinal OR Gut FlORa OR FlORa, Gut OR Gastrointestinal Microbiota OR Gastrointestinal Microbiotas OR Microbiota, Gastrointestinal OR Gut Microbiota OR Gut Microbiotas OR Microbiota, Gut OR Intestinal Microbiome OR Intestinal Microbiomes OR Microbiome, Intestinal OR Intestinal FlORa OR FlORa, Intestinal OR Intestinal Microbiota OR Intestinal Microbiotas OR Microbiota, Intestinal OR Intestinal MicroflORa OR MicroflORa, Intestinal OR Enteric Bacteria OR Bacteria, Enteric OR Gastric Microbiome OR Gastric Microbiomes OR Microbiome, Gastric) | 146678 |
| 5 | #3 OR #4 | 146678 |
| 6 | #2 OR #1 | 9818 |
| 7 | #6 AND #5 | 39 |

Supplementary table 2 Quality assessment of selected studies using the Newcastle-Ottawa Scale

| Study | Selection | | | | Comparability | | Ascertainment exposure | | | Score |
| --- | --- | --- | --- | --- | --- | --- | --- | --- | --- | --- |
|  | 1 | 2 | 3 | 4 | 5A | 5B | 6 | 7 | 8 |  |
| Ratajczak W et al. | * | * |  | * | * |  | * | * |  | 6 |
| Tsai KY et al. | * | * |  | * | * |  | * | * |  | 6 |
| Ratajczak W et al. | * |  | * | * | * |  | * | * |  | 6 |
| Takezawa K et al. | * |  |  | * | * |  | * | * |  | 5 |
| Wu S et al. | * | * | * | * | * |  | * | * | * | 8 |
| Ratajczak-Zacharko W et al. | * | * | * | * | * |  | * | * | * | 8 |

Supplementary Table 3. Alpha diversity and Beta diversity between BPH and Control

| Study | Alpha-diversity Index | Conclusion | Beta-diversity Index | Conclusion |
| --- | --- | --- | --- | --- |
| Han YY et al.2023[22] | Chao1,Shannon,Simpson | Decrease | PCoA | Distinct |
| Ratajczak W et al.2021[28] | N/A | N/A | N/A | N/A |
| Tsai KY et al.2022[29] | N/A | N/A | N/A | N/A |
| Ratajczak W et al.2023[27] | N/A | N/A | N/A | N/A |
| Takezawa K et al.2021[30] | N/A | N/A | PCoA | Distinct |
| An J et al.2023[17] | Shannon | No difference | PCoA | Distinct |
| Gu L et al.2024[19] | N/A | N/A | PCoA | Distinct |
| Gu M et al.2021[20] | N/A | N/A | N/A | N/A |
| Guo X-P et al.2023[21] | Chao1,Shannon | Increase | PCoA | Distinct |
| Li L-Y et al.2022[23] | N/A | N/A | N/A | N/A |
| Yang Y et al.2024 [24] | Chao1,ACE | Decrease | PCoA | Distinct |
| Ferrari S et al.2024[18] | N/A | N/A | N/A | N/A |
| Wu S et al.2025[25] | Chao1, ACE, Shannon, Simpson, and J indexes | Decrease | PCoA | Distinct |
| Ratajczak-Zacharko W et al[31] | Shannon, Simpso | No difference | PCoA | Distinct |
| Yang T et al[26] | N/A | N/A | N/A | N/A |

Abbreviations:BPH, benign prostatic hyperplasia.N/A, not available.
